# The interaction between ginseng and gut microbiota

**DOI:** 10.3389/fnut.2023.1301468

**Published:** 2023-11-17

**Authors:** Linxian Zhao, Mingxiu Sui, Tongbo Zhang, Kai Zhang

**Affiliations:** Department of General Surgery, The Second Hospital of Jilin University, Changchun, China

**Keywords:** ginseng, ginsenosides, gut microbiota, biotransformation, bioactivity

## Abstract

The importance of the gut microbiota to human health is attracting increasing attention. It is also involved in ginseng metabolism, mediating the bioactive metabolites of ginsenosides. In response, ginseng, known as the king of herbs, can regulate intestinal flora, including promoting probiotics and restricting the growth of harmful bacteria. Specifically, the interactions between ginseng or ginsenosides and gastrointestinal microbiota are complex. In this review, we summarized the effects of ginseng and ginsenosides on the composition of gut microbiota and discussed the gut microbiota-mediated biotransformation of ginsenosides. In particular, their therapeutic potential and clinical application in related diseases were also summarized.

## 1 Introduction

Ginseng, a perennial herb of the Panax genus of Araliaceae family, is among the most commonly used traditional medicinal herbs. Ginseng possesses various pharmacologic effects, including but not limited to anti-cancer, anti-oxidation, anti-inflammation, anti-apoptosis, anti-aging, anti-allergic effects (1). Ginseng is known to promote vitality, restore qi-blood, prolong life, and show effects against a variety of health conditions, including diabetes (2), tumors (3), respiratory diseases (4), ulcers (5), depression (6), cardiovascular disease (7), Alzheimer’s disease (8), and others. Unlike modern drugs discovered by targeting a specific protein, the traditional view holds that ginseng is capable of enhancing body by sufficiently tonifying qi-blood of spleen, lung, heart and kidney. Qi and blood were considered as two specific substance which play an essential role for the human body, and it is also the general name of the functional activities of human organs in the theory of traditional Chinese medicine (9). Qi-blood in relation to ginseng means energy and life force, which means ginseng can enhance body’s ability to against the damaging effects of stress and promote or restore normal physiological functions (10).

**Figure 1:**
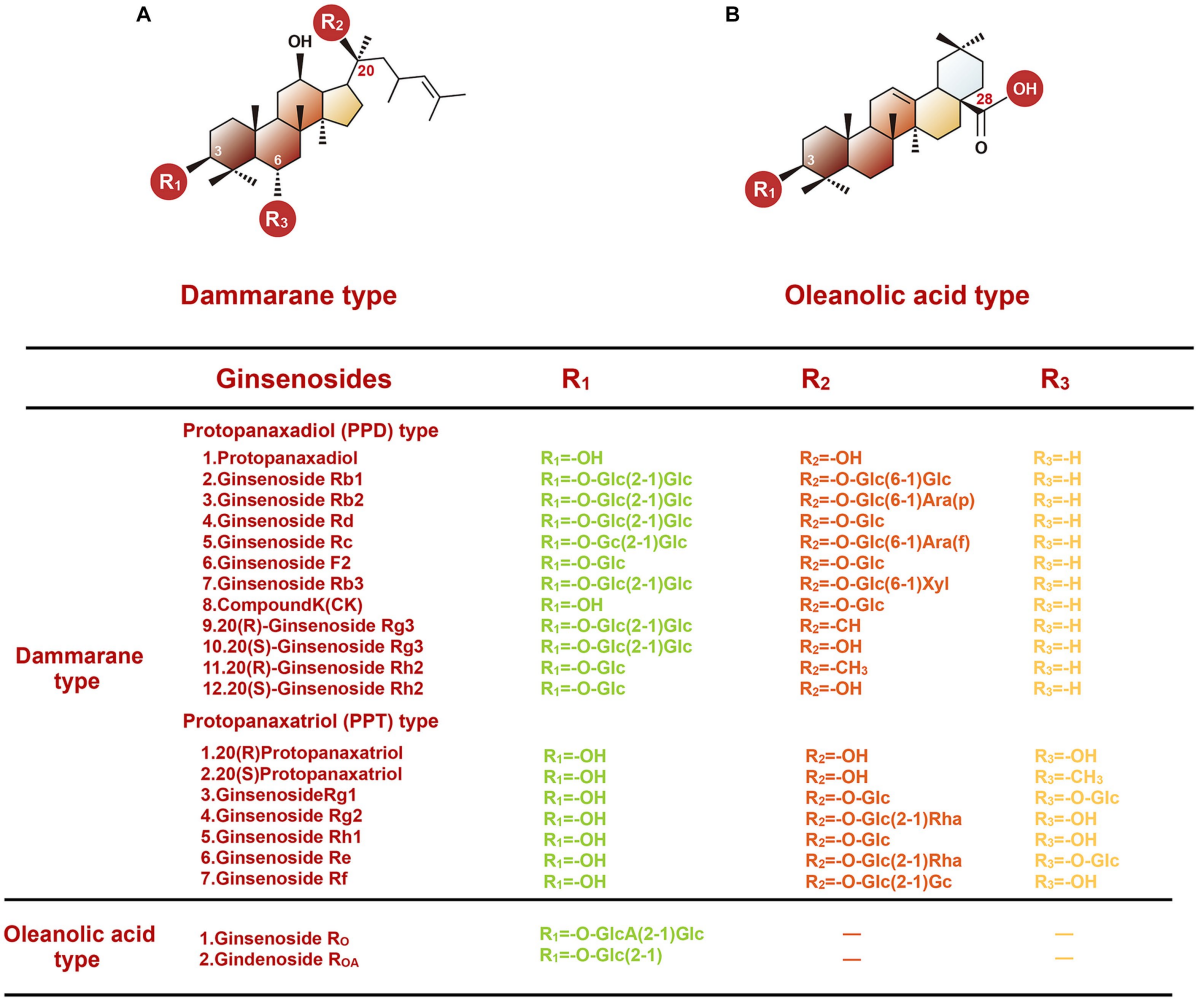
Chemical structures of two types of ginsenosides.

According to different origins, there are 11 commercially available species of ginseng. Among them, Asian ginseng, American ginseng, and Notoginseng (also named Chinese ginseng) are the three most common species of ginseng. In addition, based on the specific pharmaceutical process, ginseng can be divided into red ginseng and white ginseng. The red ginseng is usually prepared by a steaming or heating process, while the white ginseng is made by air-drying (11). To date, many active pharmaceutical ingredients have been separated from ginseng. The extracts are mainly ginsenosides, and the rest minor components include ginseng polysaccharides, ginseng polypeptides, volatile oil, cyclic peptides, and amino acids, flavonoids, trace elements, etc. (12). The most studied among them are ginsenosides, also named panaxosides, a kind of triterpene saponins found only in ginseng species. Based on the structure of aglycone skeletons, ginsenosides can be divided into dammarane and oleanolic acid types (Figure 1). The dammarane-type ginsenosides are composed of a tetracyclic ring with sugar moieties and have the most abundant tetracyclic triterpenoid saponins. The most common dammarane type ginsenosides include the protopanaxadiol (PPD) group (such as Ra1, Rb1, Rb2, Rc, Rh2), protopanaxatriol (PPT) group (such as Re, Rg1, Rg1, and F1), and ocotillol group such as majonoside R1, pseudoginsenoside F11, and vinaginsenoside R2 (13). Unlike dammarane-type, the oleanolic acid type saponins (such as Ro and R_OA_) are rare within the Panax genus. Ginsenosides, which can be produced by multiple approaches, such as high-temperature treatment, microwave treatment, enzyme treatment, and fermentation treatment. These methods can endow them with distinctly different functional and pharmacological properties. In terms of body absorption, after oral administration, the bioavailability of ginsenosides is low due to poor membrane permeability and low solubility (14). The intestinal bacteria can convert these ginseng saponins to their metabolites through hydrolyzing glycosidic bonds or stepwise cleavage of the sugar moieties, improving their biological activities.

**Figure 2:**
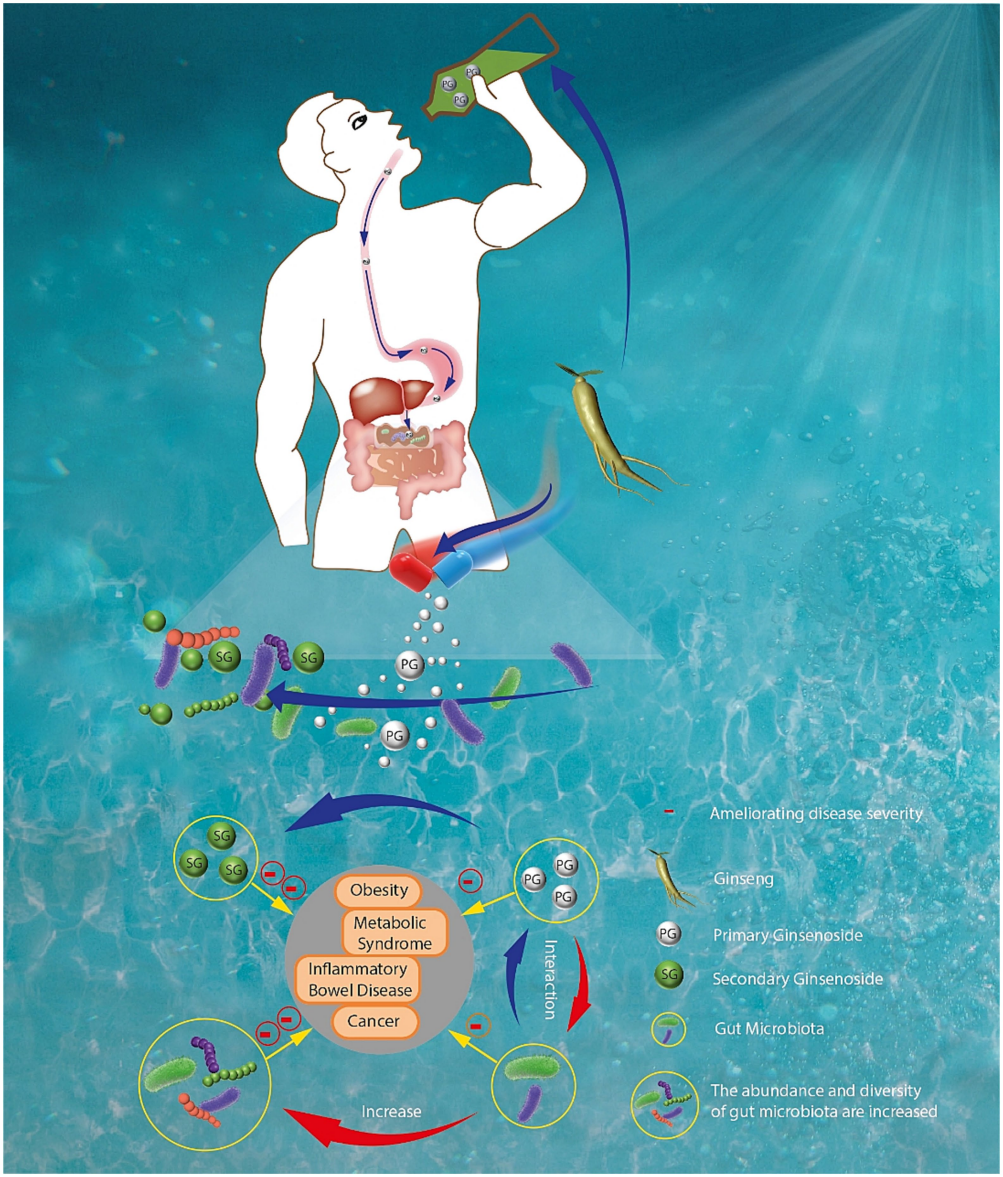
The interactions between ginseng and gut microbiota in the digestive tract.

As shown in Figure 2, the interactions between ginseng and gut microbiota are complex. Increasing studies have demonstrated that ginseng and its components play their therapeutic effects mainly through changing the gut microbiota composition and restoring gut homeostasis (15, 16). In turn, gut microbiota can transform ginsenosides, the main active components of ginseng, into rare saponins through secondary metabolites. For example, after ginseng or polar ginsenosides (such as Rb1, Rb2, and Rc) are orally administrated, they can be transformed into nonpolar bioactive ginsenosides (such as compound K, Rg3 and Rh2) by gut microbiota (17). These nonpolar ginsenosides exerted stronger pharmacologic effects than their parent ginsenosides.

## 2 Gut microbiota

To date, more than 5,000 species of bacteria in the human gastrointestinal tract have been identified, including probiotics, opportunistic pathogens, and pathogenic bacteria. They regulate intestinal function and health and influence the host’s physiological function of extraintestinal organs (18). Among these microorganisms, the most common phyla are *Actinobacteria*, *Bacteroidetes*, *Firmicutes*, and *Proteobacteria*. Especially, the population of *Firmicutes* and *Bacteroidetes* approximately represents up to 90% of total intestinal microbiota (19). Therefore, the ratio of *Firmicutes*/*Bacteroidetes* is an important indicator for evaluating the function of intestinal microbiota.

Phylum *Firmicutes* consist of over 270 genera, including obligately anaerobic and endospore-forming Gram-positive firmicutes, such as *Clostridia* and *bacilli*. Among them, the *Clostridia* class has been regarded as the most important mediator for gut microbiota, which can play two opposite roles in regulating intestinal homeostasis. On one hand, it has several beneficial roles, such as promoting host immune homeostasis and food fermentation efficiency. For example, one study proposed that *Clostridium* cluster XIVa, a major mucin-adhered microbiota, has great potential for treating inflammatory bowel disease through promoting butyrate production and enhancing butyrate bioavailability (20). On the other hand, *Clostridium perfringens* is an important pathogen of humans and domestic animals. For example, it has been verified that the human pathogen *Clostridium difficile* was the major cause of antibiotherapy-associated nosocomial diarrhoea in adults (21). In conclusion, the abundance of the phylum *Firmicutes* is closely associated with the functions of gastrointestinal tracts. For example, stool consistency of patients with constipation was related to the abundance of the *Firmicutes*.

The phylum *Bacteroidetes* include three categories: *Bacteroides*, *Prevotella* and *pooled Bacteroidetes*. Bacteroides are among the most dominant intestinal commensal bacteria in the human adult microbiota, which approximately occupies up to 25% of total intestinal microbiota (22, 23). The *Bacteroides*, with normal abundance, can promote the health of the host when they are in the right place of the gut. However, if the abundance of *Bacteroides* species is disordered or not in their proper range, they will lead to significant pathological changes. For instance, the relative abundance of *Bacteroides* species is generally lower in patients with obesity (24) and inflammatory gastrointestinal disorders (25). These findings suggested that *Bacteroides* strains may directly modulate the gut function, and their increased abundance may promote body health.

Phylum *Actinobacteria* is widely distributed in the natural environment and can also colonize most ecological niches in the human body (26). The *Actinobacteria* phyla are composed of aerobe, anaerobe, and gram stain (gram positive, gram negative) bacteria. Among them, the most important genus is *Bifidobacteria*, which can be considered probiotics due to their beneficial effects on human health. *Bifidobacteria* can produce and secrete various useful antibacterial substances and digestive enzymes, thus improving the nutrient metabolism and digestive function and maintaining gut homeostasis. Probiotics can protect the host from the invasion of various pathogens via promoting favorable immunomodulation and enhancing nutrient metabolism efficiency. The most common probiotics include *Bifidobacterium*, *Lactobacillus*, and *Enterococcus* (27).

Phylum *Proteobacteria* is a subtype of Gram-negative bacteria which belongs to the *Enterobacteriaceae* family. Enterobacteriaceae family includes a kind of very common intestinal pathogen, and its dysbacteriosis is involved in the development of various gastrointestinal disorders, especially inflammatory bowel diseases such as Crohn’s disease and ulcerative colitis (28). To date, although the special role of *Proteobacteria* in regulating intestinal homeostasis remains unclear, numerous studies have proposed that its increased abundance should be seen as an important diagnostic clue for health dysbiosis and diseases. Moreover, various common factors such as environmental changes and eating habits can change the growth and prevalence of the *Proteobacteria* (29). Therefore, a better understanding of the biological function of *Proteobacteria* will greatly help us identify the relationship between the host and microbes in the mammalian gut.

For different individuals, the composition and abundance of gastrointestinal microbiota are completely different, which may be caused by various factors, such as lifestyle habits, genetics, hormones, environmental changes, and drug influence (30). These differences also increase the difficulty of investigating the gut microbiota. In recent years, the interaction between gut microbiota and various natural drugs has drawn attention. Gastrointestinal microbiota can significantly impact biological metabolite processes and change the pharmacological functions of various drugs (31). In particular, recent evidence has demonstrated that gut microbiota is involved in the biotransformation of ginseng and ginsenosides, thus changing their pharmacological properties (32). In addition, as shown in Table 1, various studies have reported ginseng and its components have therapeutic effects on various diseases through regulating gut microbiota imbalance (55, 56). The current progress in the interaction between gastrointestinal microbiota and ginseng was summarized in the following.

**Table 1:** The effects of ginseng and its extracts on various diseases and the composition of gut microbiota.

Ginsengs types	Species	Diseases	Changes of gut microbiota		Ref.
Asian ginseng	Rat	NA	**↑***Bifidobacterium* spp. **↑***Lactobacillus* spp. **↑***Parasutterella* spp.	**↑***Allobaculum* spp. **↑***Clostridium* spp.	(33)
Asian ginseng	Mice	Obesity	**↑** *Enterococcus faecalis*		(34)
Asian ginseng	Mice	Nonalcoholic fatty liver disease	**↑** *Parabacteroides* **↑** *Muribaculaceae* **↓** *Helicobacter*	**↑** *Akkermansia* **↑** *Ruminococcus_torques* **↓** *Lachnospiraceae*	(35)
			**↑**The ratio of *Firmicutes*/*Bacteroidetes*		
Asian ginseng	Human	Obesity	**↑** *Proteobacteria* **↑** *Bifidobacterium* **↑** *Anaerostipes*	**↑** *Blautia* **↑** *Faecalibacterium*	(36)
Asian ginseng	Rat	Exercise-induced fatigue	**↑** *Lactobacillus* **↑** *Bifidobacterium* **↓** *Anaerotruncus* **↓** *Clostridium*	**↑** *Bacteroides* **↑** *Coprococcus* **↓** *Streptococcus*	(37)
Asian ginseng	Rat	Inflammation	**↑***Parasutterella* **↑***Lactobicillus* **↓**Harmful TM7	**↑** *Proteobacteria* **↑** *Methylobacteriaceae* **↑** *Sutterellaand*	(16)
American ginseng	Mice	Gut barrier dysfunctions	**↑** *Clostridiales* **↑** *Bifidobacterium* **↑** *Lachnospiracea*	**↓** *Escherichia-Shigella* **↓** *Peptococcaceae*	(19)
American ginseng	Mice	Colorectal cancer	**↑** *Firmicutes*	**↓** *Bacteroidales* **↓***Verrucomicrobia*	(21)
Panax notoginseng	Mice	Obesity	**↑** *Akkermansia muciniphila* **↑** *Parabaceroides distasonis*		(38)
Panax notoginseng	Mice	Colorectal cancer	**↑**Akkermansia spp.		(39)
White ginseng	Rat	NA	**↑**Total bacteria	**↑***Lactobacillus* strains	(40)
Red ginseng	Mice	Inflammatory bowel disease	**↑** *L. johnsonii* **↑** *L. reuteri*	**↑** *P. goldsteinii*	(41)
Red ginseng	Rat	Ulcerative colitis	**↑** *Lactobacillus*	**↑** *Bifidobacterium*	(42)
Red ginseng	Mice	Aging	**↑** *Bifidobacterium* **↑** *Akkermania*	**↓** *Desulfovibrio* **↓** *Acetatifactor*	(43)
Red ginseng	Human	Metabolic syndrome	**↑** *Bacteroidetes*	**↓** *Firmicutes* **↓** *Proteobacteria*	(44)
Red ginseng	Mice	Obesity	**↑** *Akkermansia* **↑** *Parabacteroides* **↓** *Oscillibacter* **↓** *Helicobacter*	**↓** *Barnesiella* **↓** *Bacteroides* **↓** *Allistipes* **↓** *Lactobacillus*	(45)
Fermented ginseng	Rat	Obesity	**↑** *Prevotella_9*	**↓** *Muribaculaceae*	(46)
			**↓**The ratio of *Firmicutes/Bacteroidetes*		
Fermented ginseng	Mice	Alcoholic injury	**↑** *Akkermansia* **↑** *Dorea* **↑** *Eubacterium Bilophila* **↑** *Oscillospira* **↑** *Ruminococcus*	**↑** *SutterellaAllobaculum* **↑** *Dehalobacterium* **↓** *Parabacteroides* **↓** *Unclassified S24-7* **↓** *Peptostreptococcaceae*	(47)
Fermented notoginseng	Mice	Obesity	**↑** *Akkermansia* **↑** *Dehalobacterium* **↑** *Erysipeliotrichaceae* **↑** *parpabacteroides*	**↓** *Allobaculum* **↓** *Erysipelotrichi* **↓** *Erysipelotrichale*	(48)
Rb1	Mice	Obesity	**↑***Akkermansia* spp. **↑***Actinobacteria* **↑***Verrucomicrobia*	**↓***Allobaculum* spp. **↓***Reyranella* spp. **↓***Eubacertium coprostanoligenes*	(49)
Rh4	Rat	Gut barrier disruption	**↑** *Bacteroides* **↑** *Alloprevotella* **↑** *Blautia* **↑** *Allobaculum*	**↑** *Hungatella* **↓** *Dubosiella* **↓** *Erysipelotrichales*	(50)
			**↓**The ratio of *Firmicutes*/*Bacteroidetes*		
Rk3	Mice	Gut microbiota dysbiosis	**↑** *Bacteroides* **↑** *Blautia genera*	**↑** *Alloprevotella*	(51)
			**↓**The ratio of *Firmicutes*/*Bacteroidetes*		
Ginseng polysaccharides	Mice	Cancer	**↑** *B. vulgatus*	**↑** *P. distasonis*	(17)
Ginseng polysaccharides	Mice	Diarrhea	**↑** *Lactobacillus* **↑** *Lactococcus*	**↑** *Streptococcu* **↓** *Bacteroides*	(52)
Ginseng polysaccharides	Rat	Intestinal inflammation	**↓**Gram-negative bacteria		(53)
Ginseng polysaccharides	Rat	Diarrhea	**↑** *Lactobacillus* **↓** *Bacteroides* **↓** *Streptococcus*	**↓** *Ochrobactrum* **↓** *Pseudomonas*	(54)

## 3 Effect of ginseng on gut microbiota

### 3.1 Asian ginseng

The whole ginseng extracts contain various pharmacological ingredients of ginseng, with numerous biological activities. Thus, they have been investigated in multiple disease studies. The interaction between these extracts and intestinal flora is complex, which has also attracted a lot of attention due to an in-depth understanding of the metabolic function of the gut microbiota. To understand the way ginseng extracts interact with intestinal microbiota, we summarized the existing literature, and found most studies have focused on the anti-inflammation and anti-obesity effects of ginseng and its extracts through restoring gut homeostasis. After treatment of ginseng extracts, the diversity and abundance of gut microbiota could be in the range that benefits host health. For instance, in a rat model experiment, researchers found that long-term consumption of ginseng or its extracts could effectively increase the diversity and abundance of the intestinal flora, which significantly upregulated *Bifidobacterium* spp., *Allobaculum* spp., *Lactobacillus* spp., *Clostridium* spp., and *Parasutterella* spp. All of them could enhance the host’s health (33). In addition, another study found that administration with whole ginseng extract could upregulate the relative abundance of *Enterococcus faecalis*, which is a key bacterium modulating various fatty acid metabolism and obesity effects (34). The above studies also demonstrated that the long-term intake of ginseng promotes human body health, especially for the maintenance of gut immune homeostasis.

Another study reported that whole ginseng extract could effectively alleviate high-fat-diet-induced nonalcoholic fatty liver disease symptoms by regulating gut microbiota and enhancing the gut barrier function (35). The specific mechanisms include: increasing the diversity of the bacterial community and decreasing the ratio of *Firmicutes*/*Bacteroidetes*, promoting the proliferation of beneficial bacteria such as *Parabacteroides* (OTU644) and *Muribaculaceae* (OTU619/190/137), *Akkermansia* (OTU237) and *Ruminococcus_torques_group* (OTU66), and downregulating harmful bacteria such as *Helicobacter* (OTU303) and *Lachnospiraceae*. Notably, most of these bacteria were closely associated with inflammatory response and obesity of metabolic-related diseases.

Moreover, ginseng extracts also can regulate the composition of gastrointestinal microbiota to show anti-obesity effects, which has been confirmed in previous study (57). However, the underlying mechanisms of these effects are not fully understood. Quan et al. (34) reported that ginseng extracts could increase the relative abundance of *Enterococcus faecalis*, thus reducing adiposity problems by further promoting the production of unsaturated long-chain fatty acids and myristoleic acid. The above results were obtained from animal experiments. To further study the underlying mechanism of anti-obesity effects of ginseng, Song et al. (36) conducted a human clinical trial where 10 obese middle-aged Korean women were regularly administrated with ginseng extracts for 8 weeks, and then gut microbiota composition was measured. They found the significant changes in gut microbiota, especially in the relative abundance of *Proteobacteria*, *Blautia*, *Faecalibacterium*, *Bifidobacterium*, and *Anaerostipes*. These findings suggested that gut microbiota can significantly influence the therapeutic effects of ginseng and its main constituents.

Previous studies showed that ginseng and its extracts could reinforce vital energy and restore the qi-blood (58). Based on that, many clinical and animal experiments have been conducted to investigate the curative effects of ginseng on exercise-induced fatigue (EF), a common clinical disease lacking effective treatments due to the complex pathogenesis (59). Gut microbiota is also involved in these regulatory mechanisms. For example, in a rat model of weight-loaded swimming, Zhou et al. (37) reported that water extract of ginseng could effectively ameliorate EF through moderating gut microbiota dysbiosis, including upregulating *Lactobacillus*, *Bacteroides*, *Bifidobacterium* and *Coprococcus*, while reducing *Anaerotruncus*, *Streptococcus* and *Clostridium*. Sun et al. (16) further reported that long-term intake of ginseng extracts increases the abundance of *Parasutterella*, *Proteobacteria*, *Methylobacteriaceae*, *Sutterellaand*, and *Lactobicillus*, while downregulating harmful TM7, thus influencing their biological metabolic process, anti-inflammation, and immune regulation ability.

### 3.2 American ginseng

American ginseng possesses multiple pharmacological effects and is among the most commonly used herbal medicines in the west (60). Similar to Asian ginseng, ginsenosides are the major bioactive components of American ginseng, and over 30 ginsenosides have been isolated from it (61). Zhou et al. (62) proposed that American ginseng polysaccharide and ginsenoside co-treatment can prevent cyclophosphamide-induced side effects via relieving the immune disorder and restoring the dysfunction of gut microbiota. They found American ginseng administration effectively upregulated multiple beneficial mucosa-associated bacteria (such as *Clostridiales*, *Bifidobacterium*, and *Lachnospiraceae*) and downregulated harmful ones (such as *Escherichia-Shigella* and *Peptococcaceae*). Another study found that it can be used to prevent or treat colorectal cancer, etc. (63). Enteric dysbacteriosis and inflammatory bowel disease have both been reckoned as leading causes of colorectal cancer. Wang et al. (64) further confirmed that American ginseng could attenuate colitis-associated colon carcinogenesis by restoring the metabolomic and microbiota profiles, including upregulaing *Firmicutes* and downregulating *Bacteroidales* and *Verrucomicrobia*.

### 3.3 Panax notoginseng

Panax notoginseng saponins (PNS), as one of the most effective components of Panax notoginseng (PN), have been widely applied in treating various diseases for over 400 years, especially for cardiovascular diseases (65). Similar to major ginsenosides, the oral bioavailability of PNS is also poor due to its poor membrane permeability, which has to be metabolized by gut microbiota in the gastrointestinal tract to yield novel metabolites, such as ginsenoside F1, ginsenoside Rh 2, GCK, PPT and PPD. After deglycosylated by intestinal flora, these metabolites possessed stronger bioavailability than their parent saponins. For instance, GCK showed better anti-cancer and anti-inflammatory properties, and ginsenoside Rh2 also had a good bioavailability and stronger anti-tumor and immunomodulatory effects (66). However, in terms of human gut microbiota groups driven by different diets, the metabolism profiles of PNS were significantly different (67), which might be due to the different composition of gut microbiota in these groups. In addition, the metabolites of PNS were obviously different between *in vivo* and *in vitro* experiments (66). The *in vivo* biotransformation could produce more species of PNS metabolites than *in vitro*, such as notoginsenoside K, gypenoside LI, notoginsenoside R3, and notoginsenoside R6, which can only be found in *in vivo* experiments. However, their major metabolic pathway and metabolites were the same, both via deglycosylation reaction to produce GCK and ginsenoside Rh2. It was found that the metabolic efficiency of PNS was faster *in vitro* than that *in vivo*. These findings also demonstrated that the gut microbiota diversity could influence the pharmacological effects of PNS.

The PNS has shown huge therapeutic potential in controlling body weight (68). For example, recent studies have reported that it could reduce ectopic fat accumulation and exert a hypoglycemic effect by regulating bile acid biosynthesis and enhancing antioxidative and anti-inflammatory effects (41, 69). However, PNS is hard to be resorbed by the body because of the low drug permeability, and thus, it has enough time to further contact with gut microflora (70). Zhao et al. (38) investigated the effects of PNS on adiposity and gut microbiota in a high-fat diet-induced obesity mice. They found that the gut microbiome of this rat was changed obviously, including significantly upregulating the relative abundance of *Akkermansia muciniphila* and *Parabaceroides distasonis*, both of which are predominant bacteria in the identified microbiota. These flora changes could reduce host adiposity and promote thermogenesis and beige adipocyte reconstruction through activating the leptin-AMPK/STAT3 signaling pathway.

Previous research has reported the anti-cancer effects of PNS and its metabolites. To further investigate the role of gut microbiota in anti-cancer action, Chen et al. (39) detected the changes in intestinal microbiota after PNS treatment in an intestinal inflammation-induced colorectal cancer mouse model. They found PNS treatment significantly upregulated the abundance of *Akkermansia* spp., which was negatively associated with the development of colorectal cancer.

### 3.4 White ginseng

White ginseng is the unprocessed, sundried or air-dried ginseng, with fewer ginsenosides and polyphenolics than red ginseng due to lacking high temperature steaming treatment (71). White ginseng could modulate the intestinal microbiota composition and mucin gene expression levels. After oral administration of white ginseng, the relative abundance of total bacteria and *Lactobacillus* strains was obviously increased (40), which were beneficial to host health, and could improve the bioavailability of nutrients, enhance the immune system, and promote the production of anti-microbial substances. Moreover, white ginseng could effectively upregulate the mRNA expression level of Muc2, major intestinal mucin in rats, which could effectively enhance gut barrier function and the capacity against pathogenic bacteria.

Previous studies have demonstrated that both white and red ginseng exert anti-obesity effects through ameliorating gut microbiota dysbiosis. However, to compare their anti-obesity effects, Zhou et al. (72) treated high-fat diet-fed obese mice with white and red ginseng, respectively, under equivalent conditions. The results showed that white ginseng exerted stronger anti-obesity effects as compared to red ginseng. Further study has found that carbohydrates and ginsenosides in white ginseng are potentially present more beneficial effects to the obesity-associated gut bacteria dysbiosis (72).

### 3.5 Red ginseng

Red ginseng is produced by a heat treatment that mainly includes steaming and drying processing methods. After the high-temperature treatment, it possesses higher concentrations of active ingredients (such as polysaccharides, ginsenosides, and polyphenols) (73). After oral administration, the ingredients of red ginseng extracts underwent further secondary metabolism and absorption in the gastrointestinal tract. Available evidence has confirmed that red ginseng treatment could significantly mediate the gut microbiota composition and improve the functions of the gastrointestinal tract, especially influencing the abundance of bacterial flora closely associated with the absorption of Rd and Rg3, such as Peptococcaceae, Rikenellaceae, and Hungateiclostridiaceae (74).

As an effective anti-inflammatory drug, red ginseng extract has been considered a promising candidate to treat inflammatory bowel disease. However, the specific mechanisms are incompletely understood, especially the way it interacts with gut microbiota. In a mice model with post-infectious human irritable bowel syndrome-like symptoms, Yu et al. (75) firstly reported that red ginseng extract improved gut-brain responses by increasing the proliferation of beneficial microbes (such as *L. johnsonii*, *L. reuteri*, and *P. goldsteinii*), and normalizing enteric microbiota (such as *P. goldsteinii*). Similarly, another study also reported that it was capable of effectively alleviating the symptoms of ulcerative colitis by promoting the proliferation of probiotics (such as *Lactobacillus* and *Bifidobacterium*) (42). In addition, red ginseng is also an effective anti-aging drug, and the therapeutic mechanisms may be partially achieved through regulating the composition of the intestinal flora. In a D-galactose aging mouse model, researchers found that red ginseng delayed aging process partially via increasing the diversity of probiotics (such as *Bifidobacterium* and *Akkermania*) and decreasing inflammatory bacteria (such as *Desulfovibrio*, and *Acetatifactor*) (43).

Ginseng has been used to treat metabolic syndrome for thousands of years. However, the underlying mechanism is poorly understood. Accumulating evidence indicated that gut microbiota composition is closely associated with metabolic syndrome. To investigate whether gut microbial profile could be influenced by red ginseng administration, a randomized clinical trial was conducted where 60 patients meeting the metabolic syndrome criteria were included (44). After the treatment with Korean red ginseng for 8 weeks, the symptoms of these patients were significantly improved, such as significant reductions in systolic blood pressure, and the gut microbial population was also obviously changed, and especially *Bacteroidetes* was upregulated while *Firmicutes* and *Proteobacteria* were downregulated. To further investigate the underlying mechanisms of the anti-obesity effect of Korean red ginseng, Lee et al. (45) treated high-fat diet-induced obesity mice with its extracts and observed their gut microbiome composition. They found most changes in gut microbiota were associated with obesity and diabetes, including obviously upregulating *Akkermansia* and *Parabacteroides*, together while significantly downregulating *Barnesiella*, *Bacteroides*, *Allistipes*, *Lactobacillus*, *Oscillibacter*, and *Helicobacter*.

### 3.6 Fermented ginseng

Food fermentation has been among the oldest biotechnological technology for thousand years, usually achieved by using various edible microorganisms (76). Fermented traditional Chinese medicine often exerts stronger pharmacologic effects, with lower toxicity (77). Further study on the fermentation of ginseng extracts showed that it could obviously enhance the bioavailability and bioactivity of ginsenosides (78). For example, one study proposed that fermented ginseng seeds possessed better antioxidant properties than nonfermented ginseng seeds (79). To investigate the effects of fermented ginseng on gut microbiota and immune regulation, the fermented ginseng was firstly prepared using *Lactobacillus fermentum*, and then a rat model with antibiotic-associated diarrhea and treated with fermented ginseng was established, and the results showed that the symptoms of antibiotic-associated diarrhea and colon inflammation was obviously improved through downregulating multiple immune factors. The specific roles and mechanisms may be that fermented ginseng can restore the original gut microbial environment and alleviate intestinal inflammation. After being treated with fermented ginseng, five most common gut microbes (*Enterococcus faecium*, *Bacteroides*, *Lactobacillus murinus*, *Bifidobacterium infantis*, and *Enterobacteriaceae bacterium*) gradually recover to normal status (46). In another study, Zhao et al. (47) used monascus ruber, a common edible microorganism, to ferment ginseng and investigated the effects on lipid metabolism and gut microbiota in rats fed a high-fat diet. They found that these fermented ginsengs can effectively attenuate obesity symptoms of these rats through reshaping the diversity and abundance of intestinal flora, including upregulating the abundance of *Prevotella_9*, downregulating those of *Muribaculaceae*, and *Firmicutes/Bacteroidetes*.

The fermentation process can effectively enhance the pharmacological properties of red ginseng. In a recent study, Kim et al. (80) compared the allergic rhinitis-inhibitory effects of normal and fermented red ginsengs. As expected, fermented red ginseng exerted stronger inhibiting effects with most potently reduced IL-4 expression and blood IgE levels and improved nasal allergy symptoms. Jeon et al. (81) investigated the pharmacological and functional properties of fermented red ginseng extract by lactic acid bacteria. They found lactic acid bacteria fermentation can significantly increase deglycosylated plasma metabolites, such as protopanaxadiol (PPD), protopanaxadiol (PPT), and compound K (CK).

Long-term and excessive drinking of alcohol can destroy intestinal homeostasis and barrier function, thus leading to various diseases. Previous study has demonstrated that ginseng and its extracts could improve intestinal barrier function in alcoholic animal models (82). To study whether fermented ginseng can enhance the biological activity by regulating destroyed intestinal homeostasis, Fan et al. (48) used probiotic-fermented ginseng to treat mice models with alcoholic injuries. They found the fermented ginseng could alleviate the alcoholic liver injury and disorder of the intestine by upregulating the abundance of *Akkermansia*, *Eubacterium Bilophila*, *Dehalobacterium*, *Oscillospira*, *Sutterella*, *Allobaculum*, *Dorea*, and *Ruminococcus*, and a significant downregulation of *Parabacteroides*, *unclassified S24-7*, and *unclassified Peptostreptococcaceae*. Further study also proposed that microorganism can hydrolyze ginsenosides into minor ones during fermentation. Thus, fermented ginseng exerts a stronger physiological activity (83).

The effect of fermented PN on obesity has also been investigated (84). Going further, Shin et al. (49) proposed that fermented PN by lactic acid bacteria exhibited a stronger anti-obesity function than unfermented PN. In a high-fat diet-fed mouse model, fermented PN and normal PN were administrated, respectively, and the results showed that both two compounds could change the gut microbial composition. Compared with the normal PN group, the fermented PN treatment group has upregulated *Akkermansia*, *Dehalobacterium*, *Erysipeliotrichaceae* and *parpabacteroides*, and significantly downregulated *Allobaculum*, *Erysipelotrichi* and *Erysipelotrichale*. These distinguished gut microbial compositions are the major causes of fermented PN playing stronger anti-obesity effects.

## 4 Interaction between various ginsenosides on gut microbiota

We mainly reviewed the effects of ginseng and its extracts on the composition of gut microbiota in the sections above. However, intestinal microflorae are also involved in the metabolic process of various ginsenosides.

It was confirmed that gut microbiota could secret special enzymes to promote the metabolization of ginseng through deglycosylation, oxygenation or hydrolysis reaction (85). Specifically, the *Bacteroides* and *Lactobacillus* genera are involved in deglycosylation reaction, and *Bacteroides*, *Bifidobacterium*, *Eubacterium*, *Clostridium*, *Lactobacillus*, *Peptostreptococcus*, *Fusobacterium* and *Prevotella* genera are involved in oxygenation and hydrolysis reaction (86). Notably, these metabolic reactions can effectively enhance the bioavailability of ginsenosides. One of the most representative ginsenosides is CK, which can be derived from parent ginsenosides Rb1, Rb2, Rb3, Rc and Rd through a series of gut microbiota-induced deglycosylation reactions (85). After undergoing these complex enzymatic reactions, CK shows better anti-tumor, antioxidant, anti-apoptotic, and anti-inflammatory properties than its parent ginsenosides. In the following, we discussed the existing reports about the interaction between various ginsenosides and gut microbiota.

### 4.1 Ginsenoside Rb1, Rh4, Re, Rk3 and gut microbiota

Ginsenoside Rb1 is the most abundant and active factor found in ginseng, with multiple pharmacological activities. Recent study reported that Rb1 could improve glucolipid metabolism of obese mice by regulating gut microbial composition (87). To reveal the relevant mechanism, Yang et al. (50) firstly proposed that Rb1 supplementation reshaped gut microbiota composition, including upregulating mucin-degrading bacterium *Akkermansia* spp., *Actinobacteria*, and *Verrucomicrobia*, while downregulating *Allobaculum* spp. *Reyranella* spp. and *Eubacertium coprostanoligenes*. Among these changed bacteria, the abundance of *Akkermansia* spp. was significantly associated with obesity, hypertension, glucose metabolism, gut barrier function, and host homeostasis (88).

In contrast, gut microbiota also can influence the pharmacokinetics and metabolism of Rb1. To confirm this theory, Kang et al. (89) established two gut microbiota dysbiosis animal models induced by anti-microbials and repeated restraint stress, respectively. The results showed that the concentration of Rb1 and its deglycosylation metabolites was the opposite in these two groups. Anti-microbials treated group exhibited a significantly low level of F2 and C-K, while restraint stressed rats showed a significantly higher level of Rb 1 metabolites. That was because anti-microbials treatment inhibited the proliferation of probiotic strains such as *Proteobacteria* and *Bacteroidetes*, and promoted the growth of *Firmicutes* and *Actinobacteria* (90). However, prolonged restraint stress significantly reduced the abundance of *Porphyromonadaceae* while upregulated Gram-positive and Gram-negative bacteria such as *Citrobacter rodentium* (91). These different gut microbiota composition changes may result in significant alteration of fecal moisture and short-chain fatty acids (SCFAs). concentration, which also partially illuminated the different biotransformation functions of the two different gut microbiota dysbiosis models on Rb1. Notably, the metabolic activity of ginsenoside Rb1 in the gastrointestinal tract is different between individuals because of the differences in gut microbiota composition (92). The abundance of *Ruminococcus* spp., *Bacteroides* spp. and *Bifidobacterium* spp. was the major factor influencing intestinal bacterial metabolism.

Ginsenoside Rh4, a rare triol-type saponin isolated from red ginseng and PN, possesses multiple pharmacological properties such as anti-tumor and anti-inflammatory functions (93). Another study reported that it also could modulate intestinal alterations through regulating gut microbiota and intestinal inflammation and suppressing TLR4-MyD88-MAPK signaling pathway (51). They established a microbiota perturbation rat model via antibiotic induction, and then Rh4 was administrated. The results showed that Rh4 treatment effectively improved gut barrier disruption, including promoting the proliferation of beneficial bacteria (such as *Bacteroides*, *Alloprevotella*, *Blautia*, *Allobaculum*, and *Hungatella*), inhibiting harmful bacteria (such as *Dubosiella* and *Erysipelotrichales*), and reducing the ratio of *Firmicutes*/*Bacteroidetes.*

Ginsenoside Re, is the most abundant protopanaxatriol-type ginsenoside in the ginseng berries. In a rat experiment, ginsenoside Re could undergo a sequential catalyzed deglycosylation reaction by interacting with gut microbiota and then was biotransformed into secondary metabolites such as G-Rg1, Rg2, Rh1, F1 and protopanaxatriol (94). These main metabolites of ginsenoside Re showed stronger biological activities and better pharmacokinetic properties than their parent ginsenoside. Their further studies also found that some bacteria such as *Prevotella*, *Lactobacillus* and *Bacteroides* were involved in the metabolism of ginsenoside Re, and the abundance changes of these bacteria would significantly influence the metabolic efficiency of ginsenoside Re.

Ginsenoside Rk3 is a natural heat-treated prebiotic found in ginseng, which also can regulate the gut microbiota. Chen et al. (17) reported that oral Rk3 could improve gut microbiota dysbiosis by increasing *Bacteroides*, *Alloprevotella* and *Blautia* genera and downregulating *Firmicutes*/*Bacteroidetes* ratios. Moreover, they also found that Rk3 treatment could restore intestinal barrier dysfunction by enhancing the expression of tight junction proteins and inhibiting that of inflammatory cytokine proteins (TNF-α, IL-1β, and IL-6).

### 4.2 Ginsenoside CK, Rg3, Rh2, 20(S)-PPT, 20(S)-PPD and gut microbiota

**Figure 3:**
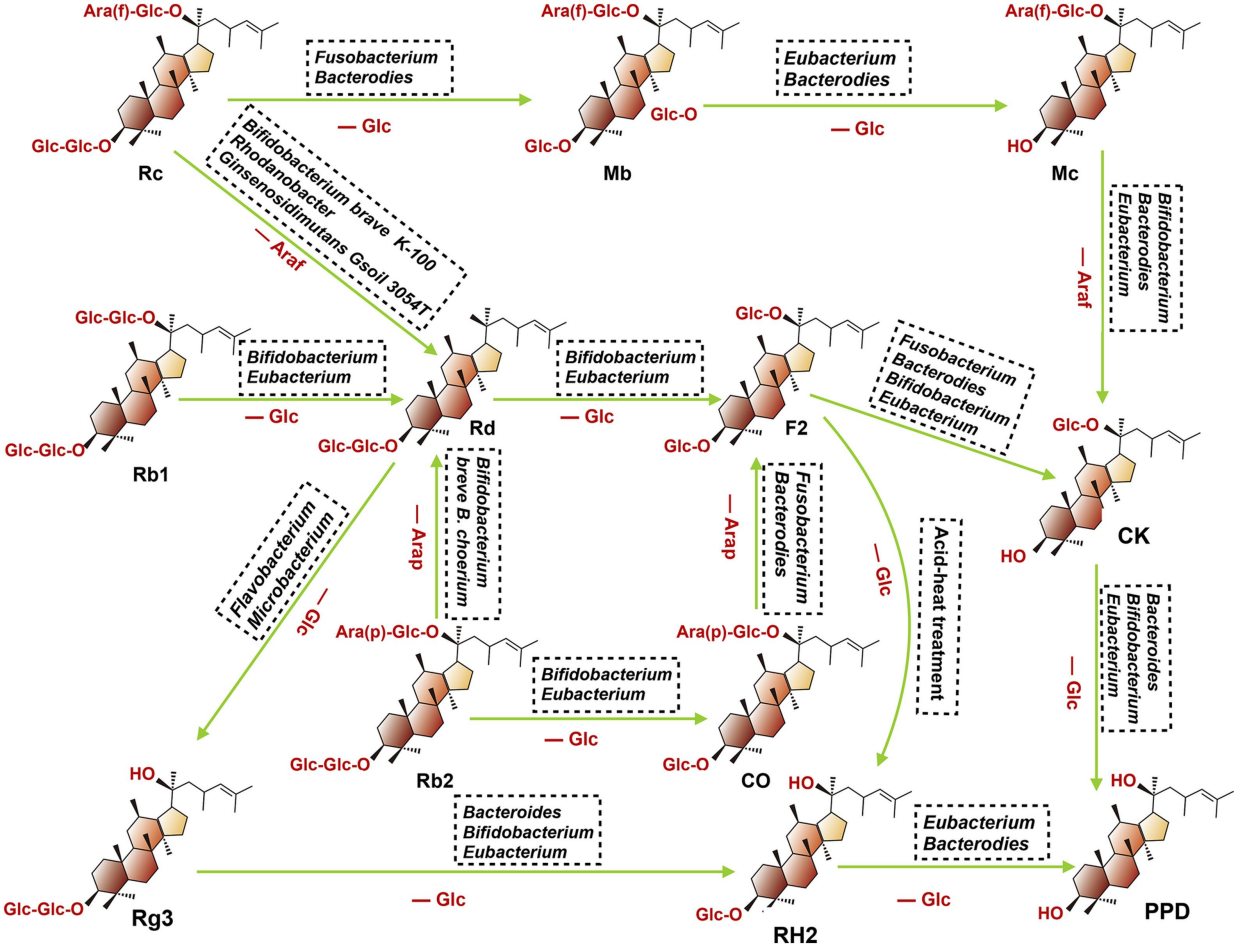
Proposed metabolism of protopanaxadiol-type ginsenoside CK, Rg3, Rh2, and PPD from the root of ginseng by gut microbiota. When fresh or dried ginsengs are orally administered in human or animals, ginsenoside Rb1, Rb2, Rc, and Rd present in these ginsengs are metabolized to compound K, Rg3, Rh2 and PPD by intestinal bacteria. (Ara(f), Arabitol(fucosyl group); Ara(p), Arabitol(phosphate group): Glc, Glucose; Rc, ginsenoside Rc; Mb, ginsenoside Mb; Mc, ginsenoside Mc; Rb1, ginsenoside Rb1; Rd, ginsenoside Rd; F2, ginsenoside F2; Rb2, ginsenoside Rb2; CO, ginsenoside CO; CK, ginsenoside CK; Rg3, ginsenoside Rg3; RH2, ginsenoside RH2; PPD, ginsenoside PPD).

After oral administration various ginsenosides, the host gut microbiota can secret multiple metabolic enzymes such as glucosidase and uronic acid enzymes, thus promoting the catabolism of primary ginsenosides through the stepwise cleavage of glycosyl or glucuronosyl moieties. To date, the major metabolites detected in the gastrointestinal tract include CK, Rg3, Rh2, 20(S)-protopanaxatriol [20(S)-PPT], and 20(S)-protopanaxadiol [20(S)-PPD]. They were discussed at length in the following (Figure 3).

Ginsenoside CK, also named 20-O-beta-D-glucopyranosyl-20(S)-protopanaxadiol M1, can be transformed into Rb1, Rb2, Rb3, Rc and Rd in the gastrointestinal tract through the biotransformation by gut microbiota. To date, multiple microbiota species have been reported involved in these biotransformation regulations, including *Fusobacteruim*, *Bifidobacterium*, *Rhodanobacter*, *Bacteroides*, etc. For example, Park et al. (95) found that Rb1 could be predominantly converted to CK through the deglycosylation effect of *Fusobacteruim* sp., and prebiotics could enhance their bioconversion efficiency via selectively promoting the proliferation of certain bacterial stains with glycoside hydrolysis capacity (96, 97). In addition, another study reported the capacity of microbiota to convert Rb1 to CK could also be enhanced by Daikenchuto (TU-100), a pharmaceutical-grade Japanese traditional medicine, which can shape gut microbiota architecture (98).

Based on the position of C20, ginsenoside Rg3 can be divided into 20(R)-Rg3 and 20(S)-Rg3, and the latter is a stereoisomer of the former. Ginsenoside Rg3 is a steroidal saponin derived from the secondary degradation of diol saponins (such as Rb1, Rb2 and Rc). Gut microbiota plays an important role in this progress since it changed the structure of these ginsenosides via cleaving key sites of C-2 glycosidic. For instance, ginsenoside Rb1or Rd could be converted to the metabolic product 20(S)-Rg3 by the genus *Microbacterium* sp. GS514, which is a process of the consecutive hydrolysis of the terminal and inner glucopyranosyl moieties at the C-20 carbon (99). Ginsenoside Rb1 also can be transformed into Rg3 under the regulation of endophytic bacteria, *Flavobacterium* sp. GE 32, and this biological microbial hydrolysis method is attracting more attention because of its high efficiency and mild conditions (100). The Rh2 is a further metabolic product of Rg3, which is produced when Rg3 loses the C-2 glycosyl group, and multiple gut bacteria species are involved in this progress, including *Bacteroides*, *Eubacterium* and *Bifidobacterium* (101).

**Figure 4:**
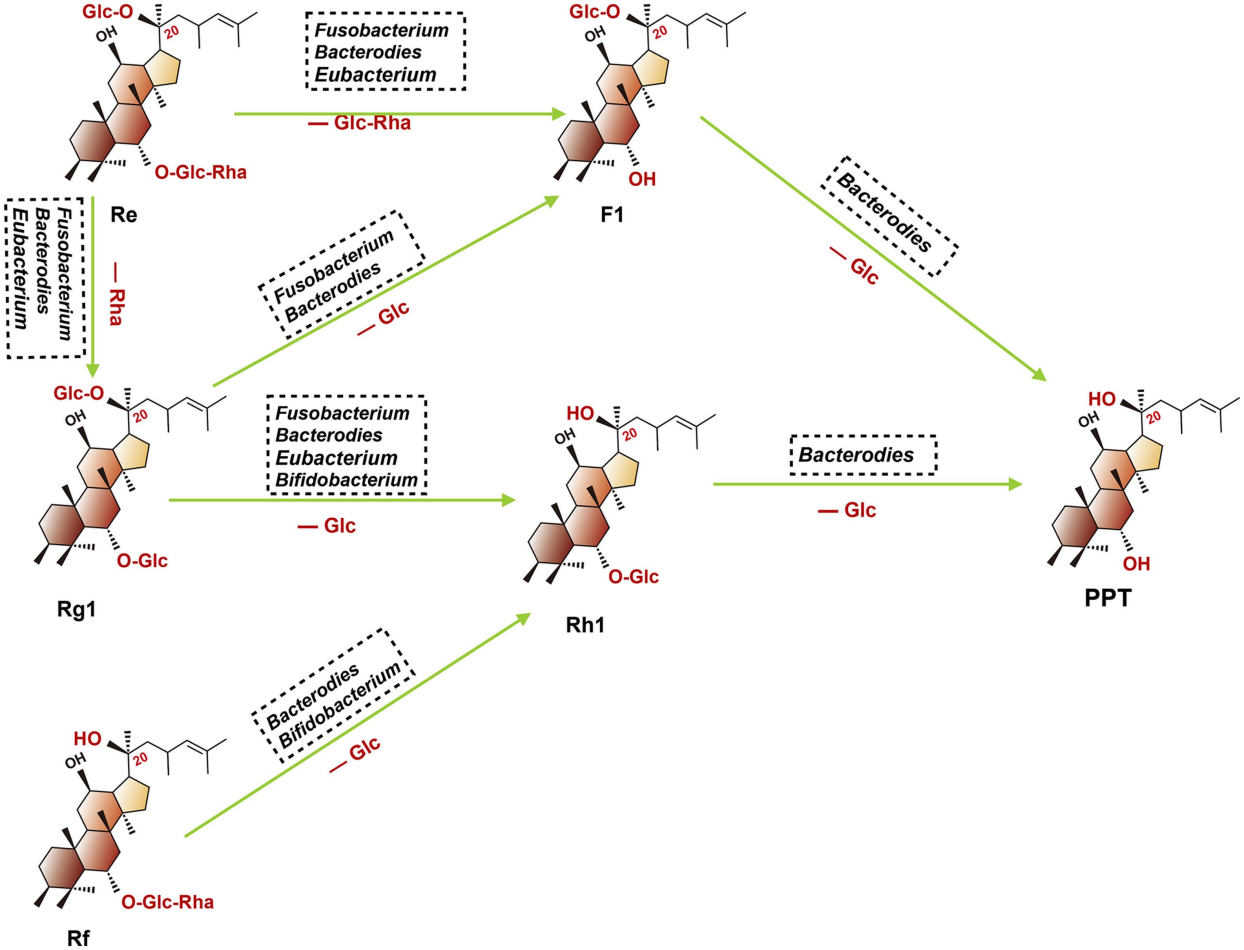
Proposed metabolism of protopanaxatriol ginsenoside Re, Rg1, and Rf from dried ginseng by gastrointestinal microbiota. When fresh or dried ginsengs are orally administered in humans or animals. Re, Rg1, and Rf in these ginsengs are metabolized to PPT. (Glc, glucose; Rha, rhamnose; Re, ginsenoside Re; F1, ginsenoside F1; Rg1, ginsenoside Rg1; Rh1, ginsenoside Rh1; PPT, ginsenoside PPT; Rf, ginsenoside Rf).

The 20(S)-PPT was seen as the final metabolite of multiple ginsenosides (such as Re, Rg1, and Rf). As shown in Figure 4, after oral administration of these ginsenosides, a stepwise deglycosylation action occurred in the digestive tract, and these reactions were mainly regulated by gut microbiotas (such as *Bacteroides*, *Bifidobacterium*, *Eubacteroium*, and *Fusobacterium*) (102). The 20(S)-PPD is a dammarane-type tetracyclic terpene sapogenin produced mainly via two metabolic pathways, as shown in the following (103). Firstly, ginsenoside CK, derived from Rb1, Rb2 and Rc, which can be further metabolized to 20(S)-PPD by gut microbiota (such as *Bacteroidetes*, *Bifidobacterium* and *Eubacterium*). For the second pathway, ginsenoside Rg3 was firstly concerted into Rh2, then further transformed into 20(S)-PPD by *Bacteroides* and *Eubacterium*.

## 5 Polysaccharides and gut microbiota

Ginseng polysaccharides (GPs), as a representative active ingredient of ginseng, can modulate immunopotentiation function, improve intestinal metabolism, alleviate inflammation and oxidative stress, enhance anti-cancer activity, and especially regulate gut microbiota (6, 104). Among these biological activities, reshaping the intestinal flora may be a future research focus. Previous studies have reported that GPs could increase the relative abundance of *Lactobacillus* spp. and *Bacteroides* spp., thus changing the gut microbial environment and enhancing the absorption of ginsenosides (105, 106).

A recent study proposed that GPs could improve the anti-cancer response to programmed death 1 and its ligand 1 (PD1/PD-L1) immunotherapy by reshaping gut microbiota and downregulating kynurenine/tryptophan ratio (52). After the administration of GPs combined with αPD-1 monoclonal antibody, the relative abundance of *B. vulgatus* and *P. distasonis* were obviously upregulated, and they were over-represented among Chinese non-small cell lung cancer with anti-PD-1 blockade responders. In addition, in a mice model experiment with antibiotic-associated diarrhea, GPs effectively alleviated the symptoms of diarrhea by reshaping gut microbial environment, including upregulating the abundance of *Lactobacillus*, *Lactococcus*, and *Streptococcus*, while reducing that of *Bacteroides* (53).

Wang et al. (54) proposed that polysaccharide could play intestinal anti-inflammatory effects through modulating intestinal microbiota and mTOR-dependent autophagy pathway. In an experimental model of rats with intestinal inflammation induced by dextran sulfate sodium, they found polysaccharide administration obviously reduced the abundance of Gram-negative bacteria. Thus, the activity of lipopolysaccharide, an essential component of the cell wall of Gram-negative bacteria, and the expression of TLR4, the receptor lipopolysaccharide, were inhibited. In addition, in an antibiotic-associated diarrhea rat model induced by gastric gavage with lincomycin hydrochloride, the administration of ginseng neutral polysaccharide could alleviate diarrhea symptoms by influencing gut microbiota composition, including obviously upregulating the abundance of *Lactobacillus*, and downregulating the genus level of *Bacteroides*, *Streptococcus*, *Ochrobactrum* and *Pseudomonas* (107).

## 6 Discussion and conclusion

Gut microbiota is described as the third organ, including hundreds of microbial species. Remarkably, the number of microbial cells in the gut is roughly equivalent to that of somatic cells in the human body (108). As the foremost and most diverse microbial community, gastrointestinal microbiota could influence host health with several beneficial effects, such as protection against pathogens, immune regulation, food fermentation, production of vitamins B and K, and promoting biological metabolism (109). The metabolic activity of gut microbiota could significantly influence the pharmacological effects of various drugs. With an enhanced understanding of the biological function of gut microbiota, increasing studies showed that it was involved in the metabolic function of ginseng and its extracts. Oral administration is the most common intake method for ginseng and its extracts. However, cell membranes with poor permeability make it difficult for them to be absorbed by the human body, thus limiting their pharmacological activities. After oral administration, most primary ginsenosides (such as Rb1, Rd, and Rg1) are converted to deglycosylated metabolites (such as CK, Rh1, and F1) by gut microbiota, and these secondary ginsenosides possess stronger pharmacological properties than their parent ginsenosides (110). Intestinal microbiota can produce some special intestinal enzymes to promote the hydrolyzing of glycosidic linkage and biotransformation of various saponins. During these biotransformation progress, the most important bacteria is *Bifidobacterium*, which is involved in most biotransformation of ginsenosides *in vivo*. It is also among the most common probiotics and can enhance intestinal immune function and increase bioavailability and drug efficacy. In terms of different individuals, the composition of intestinal microbiota is completely different, leading to significant variation in their metabolic activities.

In conclusion, most of the research remains at the superficial-level. More studies should be conducted to investigate the interactions between ginseng and gut microbiota on the cellular and molecular-level. The studies on the underlying mechanisms for homeostasis maintenance or restoration are warranted.

## Author contributions

LZ: Conceptualization, Project administration, Writing – original draft. TZ: Data curation, Writing – original draft. KZ: Conceptualization, Project administration, Writing – review & editing. MS: Methodology, Writing – review & editing.
